# Functional imaging with dual-energy computed tomography for supplementary non-invasive assessment of mast cell burden in systemic mastocytosis

**DOI:** 10.1038/s41598-022-18537-6

**Published:** 2022-08-20

**Authors:** Julia Riffel, Johannes Lübke, Nicole Naumann, Sebastian Kreil, Georgia Metzgeroth, Alice Fabarius, Karl Sotlar, Hans-Peter Horny, Mohamad Jawhar, Daniel Overhoff, Stefan Schoenberg, Wolf-Karsten Hofmann, Thomas Henzler, Juliana Schwaab, Andreas Reiter, Philipp Riffel

**Affiliations:** 1 Institute of Clinical Radiology and Nuclear Medicine, University Hospital Mannheim, Heidelberg University, Mannheim, Germany; 2 Department of Hematology and Oncology, University Hospital Mannheim, Heidelberg University, Theodor-Kutzer-Ufer 1-3, 68167 Mannheim, Germany; 3 Department of Pathology, Paracelsus Medical University of Salzburg, Salzburg, Austria; 4 Department of Pathology, Ludwig-Maximilians-University, Munich, Germany

**Keywords:** Myeloproliferative disease, Neoplasm staging

## Abstract

Systemic mastocytosis (SM) is characterized by multifocal accumulation of neoplastic mast cells (MCs), predominately affecting the bone marrow (BM). Imaging with computed tomography (CT) is used for assessment of bone mineral density and structure. However, the value of functional imaging with dual-energy CT (DECT) and the assessment of virtual-non-calcium attenuation values (VNCa-AV) for visualization of BM disease burden in SM has not yet been assessed. DECT of the axial skeleton was performed in 18 patients with SM (indolent SM [ISM], n = 6; smoldering SM [SSM]/advanced SM [AdvSM], n = 12) and 18 control subjects. VNCa-AV were obtained in 5 representative vertebraes per patient and correlated with laboratory, morphologic and molecular parameters. VNCa-AV strongly correlated with quantitative BM MC infiltration (r = 0.7, R^2^ = 0.49, *P* = 0.001) and serum tryptase levels (r = 0.7, R^2^ = 0.54, *P* < 0.001). Mean VNCa-AV were significantly higher in SSM/AdvSM as compared to ISM (− 9HU vs. − 54HU, *P* < 0.005) and controls (− 38HU, *P* < 0.005). Nine of 10 (90%) patients with a VNCa-AV >  − 30HU and 7/7 (100%) patients with a VNCa-AV >  − 10HU had SSM or AdVSM. BM VNCa-AV provide information about the MC burden of SM patients and correlate with SM subtypes. DECT may therefore serve as a supplementary tool for SM diagnosis, subclassification and monitoring in a one-stop-shop session.

## Introduction

Systemic mastocytosis (SM) is a rare hematologic neoplasm characterized by neoplastic expansion and accumulation of clonal mast cells (MCs), predominantly affecting bone marrow (BM), skin and visceral organs^1–5^. SM is subcategorized in indolent SM (ISM), smoldering SM (SSM) and advanced SM (AdvSM), the latter comprising aggressive SM (ASM), SM with an associated hematologic neoplasm (SM‐AHN) and mast cell leukemia (MCL)^6^. While ISM patients benefit from an almost normal life expectancy, AdvSM is associated with poor prognosis and a median overall survival of less than 3–4 years^1,7–14^.

BM histology with qualitative and quantitative assessment of MC and AHN is of fundamental importance for diagnosis and subclassification of SM. In addition to blood counts and serum chemistry, radiological imaging including X-ray, computed tomography (CT) and magnetic resonance imaging (MRI) are pivotal supplementary tools for visualizing anatomical disease extent and specifically bone and BM involvement^9,15–18^. In a recent study on measurement of bone mineral density (BMD) through CT, an increased BMD/osteosclerosis was associated with a more aggressive phenotype and inferior survival^19^. Moreover, an activated BM, reflected as BM edema in MRI, is indicative for a high MC burden, organ damage and adverse survival^20^. However, BM edema can only be visualized but not quantified by MRI and decreased/increased BMD can only be quantified by CT.

To overcome those individual limitations of CT and MRI, functional imaging through dual-energy CT (DECT) and analysis of virtual-non-calcium attenuation values (VNCa-AV) has recently been established for detection of BM edema in trauma patients^21,22^ and discrimination between various infiltration patterns in multiple myeloma^23^. We therefore sought to investigate whether functional imaging with DECT may also be useful in patients with SM for visualization of BM disease burden.

## Methods

### Patients and control group

This retrospective analysis included 18 patients with SM (female, n = 8 [44; median age 63 years, range 45–86). Detailed demographic and SM-associated disease characteristics are presented in Tables 1 and 2. Eighteen control patients (female [n = 7, 39%]; median age 61 years, range 41–83) were included using the same DECT protocol. All control patients were diagnosed with non-metastatic malignant melanoma; other hematologic neoplasms were not present. The analysis adhered to the tenets of the Declaration of Helsinki and was approved by the relevant institutional review board (Medical Faculty Mannheim, University of Heidelberg). All patients gave written informed consent.

**Table 1:** Demographical and disease characteristics of 12 patients with smoldering or advanced systemic mastocytosis.

#	Sex	Age in years at Dx	WHO Dx	Type of AHN	Time from Dx to DECT (years)	VNCa values (HU)	A/T	Serum tryptase (µg/L)	MCI in BM (%)	*KIT* D816V EAB in BM (%)	*KIT* D816V EAB in PB (%)	Other mutations	Karyotype
1	m	50	SSM	–	7, 24	− 1	−/−	545	20	14	14	–	46, XY
2	m	56	ASM	–	0, 76	6	−/−	194	35	45	18	–	46, XY
3	m	66	SM-AHN	MDS/MPN-U	0, 66	− 6	−/+	206	20	42	46	ASXL1, SRSF2, TET2	46, XY
4	f	42	SSM	–	3, 42	7	−/−	302	70	42	0	–	46, XX
5	f	81	SM-AHN	MDS/MPN-U	1, 34	24	+/+	554	60	16	54	TET2	46, XX
6	m	72	SM-AHN	MDS/MPN-U	1, 19	5	−/−	377	35	8, 4	18	JAK2, SRSF2	46, XY
7	m	65	SM-AHN	MDS/MPN-U	0, 86	2	−/−	430	50	29	22	ASXL1, TET2	46, XY
8	m	54	MCL	–	0, 33	7	−/−	885	70	*KIT* neg	*KIT* neg	–	46, XY
9	f	63	SM-AHN	MDS	0, 73	− 64	+/−	87	25	23	0	–	46, XX
10	f	55	SSM	–	5, 75	− 40	−/−	184	30	*KIT* neg	*KIT* neg	–	46, XX
11	m	66	SM-AHN	MPNeo	0, 50	− 20	−/−	146	5	14	8, 1	*JAK2*	47, XY, + 19
12	m	55	SM-AHN	CMML	1, 42	− 22	+/−	155	40	37	41	*SRSF2*	46, XY

Data obtained at time of DECT. A blank row separates the patients with either pathologically elevated VNCa values (n = 8, at the top) or normal VNCa values (n = 4; at the bottom).

*AHN* associated hematologic neoplasm, *A/T* anemia < 10.0 g/dL (+), > 10.0 g/dL (−), platelets < 100 × 10^9^/L (+), > 100 × 10^9^/L (−), *BM* bone marrow, *CMML* chronic myelomonocytic leukemia, *DECT* dual-energy CT, *Dx* diagnosis, *EAB* expressed allele burden, *f* female, *m* male, *MCI* mast cell infiltration, *MDS* myelodysplastic syndrome, *MDS/MPN-U* myelodysplastic/myeloproliferative neoplasm, unclassified, *MPNeo* myeloproliferative neoplasm with eosinophilia, *PB* peripheral blood, *SM* systemic mastocytosis, *SSM* smoldering systemic mastocytosis, *VNCa* virtual-non-calcium attenuation, *WHO* World Health Organization.

**Table 2:** Demographical and disease characteristics of 6 patients with indolent systemic mastocytosis.

#	Sex	Age in years at Dx	WHO Dx	Time from Dx to DECT (years)	VNCa values (HU)	A/T	Serum tryptase (µg/L)	MCI in BM (%)	*KIT* D816V EAB in BM (%)	*KIT* D816V EAB in PB (%)	Other mutations	Karyotype
13	m	58	ISM	0, 67	− 59	−/−	15	5	Not done	Not done	–	46, XY
14	m	41	ISM	1, 41	− 74	−/−	31	10	0, 8	0, 8	–	46, XY
15	f	56	ISM	0, 00	− 62	−/−	29	10	3	0, 9	–	46, XX
16	m	64	ISM	0, 75	− 15	+/−	46	10	12	21	–	46, XY
17	f	54	ISM	5, 34	− 80	−/−	20	5	20	0, 8	–	46, XX
18	m	56	ISM	11, 01	− 35	−/−	162	15	10	15	–	46, XY

Data obtained at time of DECT. All patients presented with normal VNCa values.

*AHN* associated hematologic neoplasm, *A/T* anemia < 10.0 g/dL (+), > 10.0 g/dL (−), platelets < 100 × 10^9^/L (+), > 100 × 10^9^/L (−), *BM* bone marrow, *CMML* chronic myelomonocytic leukemia, *DECT* dual-energy CT, *Dx* diagnosis, *EAB* expressed allele burden, *f* female, *m* male, *MCI* mast cell infiltration, *MDS* myelodysplastic syndrome, *MDS/MPN-U* myelodysplastic/myeloproliferative neoplasm, unclassified, *MPNeo* myeloproliferative neoplasm with eosinophilia, *PB* peripheral blood, *SM* systemic mastocytosis, *SSM* smoldering systemic mastocytosis, *VNCa* virtual-non-calcium attenuation, *WHO* World Health Organization.

### Diagnosis and subclassification

Diagnosis and subclassification were performed according to the revised world health organization (WHO) 2017 classification: ISM in 6/18 (33%) and SSM/AdvSM in 12/18 (67%) patients (Tables 1, 2)^1,2,24^. Diagnosis of SSM was established through presence of at least two of three B-findings (MC infiltration > 30% and serum tryptase level > 200 ng/mL; signs of dysplasia or myeloproliferation in non-MC lineage compartments of the BM, but no AHN; hepatomegaly without impairment of liver function and/or splenomegaly and/or lymphadenopathy). Diagnosis of ASM was based on the presence of one or more C-findings (cytopenia with neutropenia < 1 × 10^9^/L, anemia <10 g/dL or thrombocytopenia < 100 × 10^9^/L, palpable hepatomegaly with impaired liver function, palpable splenomegaly with signs of hypersplenism, malabsorption with weight loss due to gastrointestinal MC infiltrates or skeletal involvement with large osteolytic lesions and/or pathological fractures). SM-AHN met the criteria for SM and for an AHN (e.g. chronic myelomonocytic leukemia, myelodysplastic/myeloproliferative neoplasm unclassified [MDS/MPNu] or chronic eosinophilic leukemia). MCL was diagnosed based on the presence of at least ≥ 20% MCs in a BM smear.

### Treatment

Patients with ISM received a symptom-directed conventional therapy including H1- and H2-antagonists, cromolyn acid, proton pump inhibitors and corticosteroids. One patient with SSM was treated with hydroxurea. All patients with AdvSM were treated with the multikinase inhibitor midostaurin; 3 patients further received the purine analogue cladribine as second-line treatment. The use of bisphosphonates was not documented in any patient at time of DECT.

### DECT scan protocol, image reconstruction and postprocessing

All examinations were performed on a dual-source CT system in dual-energy mode (SOMATOM Force; Siemens Healthineers, Forchheim, Germany). Median time between diagnosis and DECT was 1.0 years (range 0–11.0). For evaluation of osteosclerosis, weighted-average coronal and sagittal multiplanar reformations were calculated. For DECT postprocessing, axial slices with a thickness of 1.0 mm were reconstructed. Postprocessing was performed on a dedicated dual-energy software (Syngo.via; version VB30A; Siemens Healthineers) with a three-material decomposition algorithm for bone mineral, yellow marrow and red marrow^25^. For further assessment, DECT images were viewed as weighted-average CT merged with a colour-coded VNCa overlay using the BM setting (Siemens Healthineers).

### CT image evaluation

#### Qualitative image analysis of osteosclerosis

CT images were reviewed in consensus by two attending radiologists with 10 years of experience each in body imaging on a commercially available MacPro workstation (Apple, Cupertino, CA) running OsiriX DICOM Viewer 64-bit Version 5.5.2 (OsiriX Foundation, Geneva, Switzerland) without knowledge of the clinical findings or classification. Based on our prior experience, three distinct patterns were defined: normal bone structure, diffuse osteosclerosis and multiple focal sclerotic bone lesions.

#### Quantitative image analysis

All images were separately analyzed in a randomized order by two readers (J.R. and P.R.) with 10 years of experience each in oncologic imaging. The readers were blinded to clinical data and VNCa-AV measurements were performed in consensus. In patients with SM and patients from the control group, five circular region of interest (ROI) measurements of at least 100 mm^2^ were obtained between Th11–12 and L1–3. ROI borders were maintained 2 mm away from adjacent cortical bone in order to only include BM in the evaluation. Non-myelomatous lesions, such as Schmorl nodes and haemangiomas were not included in the ROIs. For further analysis, mean values of the five ROIs were used.

### Statistics

All statistical analyses considered clinical and laboratory parameters obtained at time of imaging. The Mann–Whitney U test was used to compare continuous variables and medians of distributions. Pearson’s correlation coefficient was used to compare VNCa-AV with various disease-specific parameters (e.g. BM MC infiltration, serum tryptase level). P-values of < 0.05 (two-sided) were considered as significant. SPSS version 22 (IBM Corporation, Armonk, NY, USA) was used for statistical analysis.

## Results

### Qualitative image analysis of osteosclerosis

**Figure 1:**
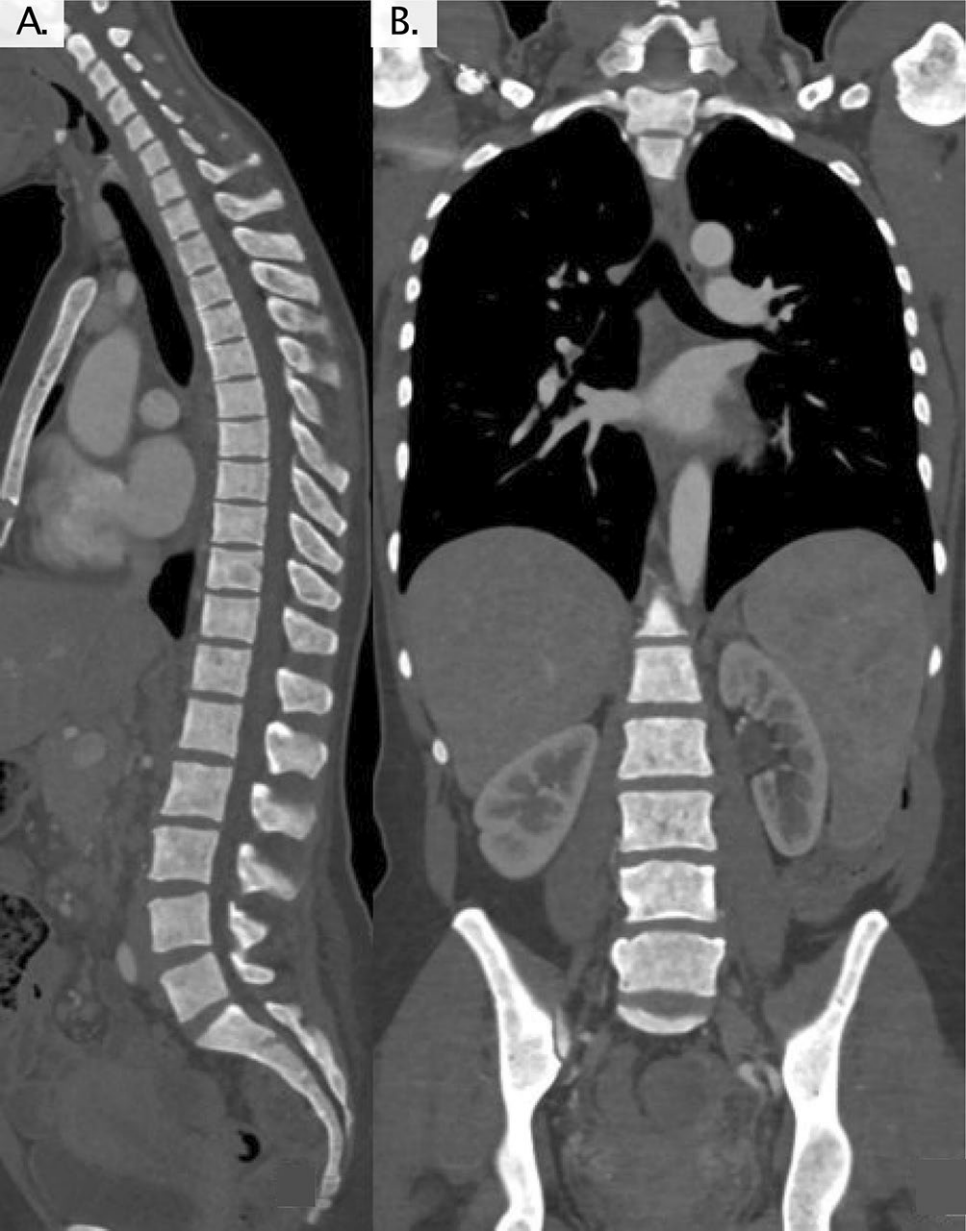
(**A**) Sagittal and (**B**) frontal view of diffuse osteosclerosis in patient #4 diagnosed with smoldering systemic mastocytosis.

Conventional CT revealed normal bone structure in all patients of the control group (18/18) and in 5/6 (83%) patients with ISM. ISM patient #18 showed multiple focal sclerotic bone lesions. A normal bone structure was identified in only 2/12 (17%) SSM/AdvSM patients, while 6/12 (50%) patients showed diffuse osteosclerosis (Fig. 1) and 4/12 (33%) patients revealed multiple focal sclerotic bone lesions.

### Quantitative image analysis of VNCa-AV

**Figure 2:**
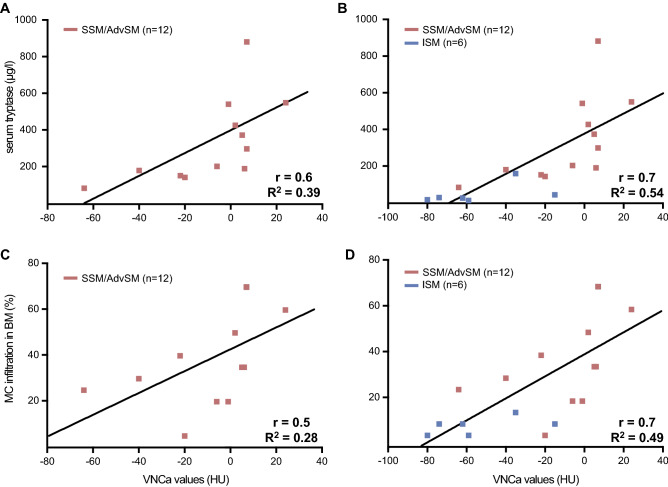
Correlation between virtual non-calcium (VNCa-AV) values and serum tryptase (**A**) and between VNCa-AV values and mast cell (MC) infiltration in bone marrow (BM) histology (**B**) in patients with smoldering/advanced systemic mastocytosis.

In total, 180 ROIs were evaluated: 90 in control subjects, 30 in ISM patients and 60 in AdvSM patients. VNCa-VA were strongly correlated with quantitative bone marrow mast infiltration (r = 0.7, R^2^ = 0.49, *P* = 0.001) and serum tryptase levels (r = 0.7, R^2^ = 0.54, *P* < 0.001). Further significant correlations were identified upon the *KIT* D816V allele burden in peripheral blood (r = 0.6, R^2^ = 0.4, *P* = 0.016) and hypoalbuminemia (r = − 0.5, R^2^ = 0.3, *P* = 0.041) (Table 3, Fig. 2). The overall results indicated a strong association between a pathologically elevated VNCa-AV and an advanced phenotype.

**Table 3:** Pearson correlation of virtual-non-calcium attenuation values with several key disease parameters in systemic mastocytosis.

Characteristics	SSM/AdvSM		ISM and SSSM/AdvSM	
	Pearson r (R^2^)	*P*	Pearson r (R^2^)	*P*
Hemoglobin	0.5 (0.13)	0.134	− 0.3 (0.08)	0.241
Platelets	0.0 (0.00)	0.988	− 0.4 (0.20)	0.065
Serum tryptase	**0.6 (0.39)**	**0.029**	**0.7 (0.54)**	** < 0.001**
Albumin	− 0.1 (0.02)	0.696	** − 0.5 (0.25)**	**0.041**
Alkaline phosphatase	0.2 (0.03)	0.582	0.3 (0.11)	0.183
Bilirubin	0.1 (0.01)	0.734	0.2 (0.05)	0.399
Mast cell infiltration in BM	0.5 (0.28)	0.079	**0.7 (0.49)**	**0.001**
*KIT* D816V EAB in PB	0.4 (0.17)	0.231	**0.6 (0.37)**	**0.016**
*KIT* D816V EAB in BM	0.0 (0.00)	0.940	0.4 (0.11)	0.122

*BM* bone marrow, *EAB* expressed allele burden, *PB* peripheral blood.

Significant values are in bold.

**Figure 3:**
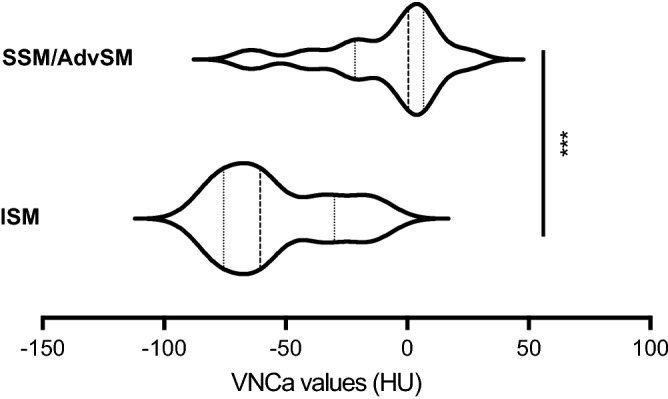
Violin plots of dual-energy computed tomography virtual non-calcium HU values (VNCa-AV) in systemic mastocytosis. Mean VNCa-AV were significantly higher in patients with SSM/AdvSM compared to ISM (− 9HU vs. − 54HU, *P* < 0.005).

**Figure 4:**
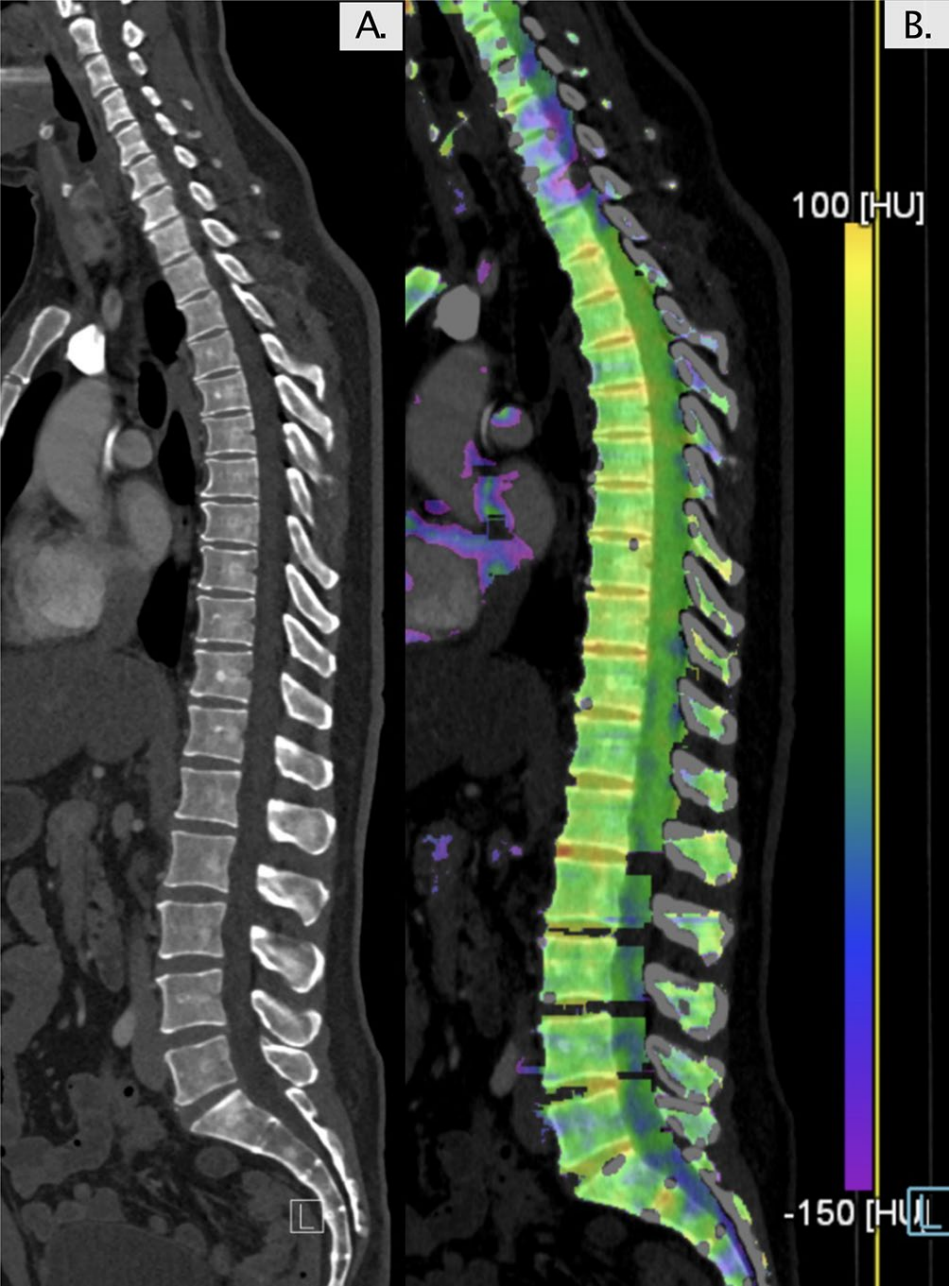
(**A**) Patient #8 with MCL showing normal bone structures on computer tomography (CT). (**B**) Dual-energy CT revealed elevated virtual-non-calcium values with diffuse edema of the bone marrow. The high bone marrow mast cell infiltration (70%) and high serum tryptase levels (885 µg/L) indicates a high mast cell disease burden.

Mean VNCa-AV were significantly higher in patients with SSM/AdvSM compared to ISM (− 9HU vs. − 54HU, *P*<0.005) and controls (− 38HU, *P* < 0.005). VNCa-AV were not different between ISM and control subjects (Fig. 3). Nine of 10 (90%) patients with a VNCa-AV > − 30HU and 7/7 (100%) patients with a VNCa-AV > − 10HU had SSM or AdVSM. Pathologically elevated VNCa-AV indicating diffuse BM edema were not observed in ISM patients (0/6) but in 8/12 (67%) SSM/AdvSM patients. Of note, two of these patients (#6 and #8) with an aggressive phenotype (BM MC infiltration: 35% and 70%, serum tryptase: 377 µg/L and 885 µg/L, genetics: *KIT* D816V+/*SRSF2*+ and *KIT* D816V negative) had normal bone structures on conventional CT (Fig. 4).

### Comparative analysis of increased and normal VNCa-AV throughout all SM subgroups

Within the cohort of SSM/AdvSM, patients with increased VNCa-AV (group 1) presented with a significantly stronger BM MC infiltration (mean 45% vs. 25%, *P* = 0.088) and higher levels of serum tryptase (mean 437 µg/L vs. 143 µg/L, *P* = 0.008), alkaline phosphatase (mean 339 U/L vs. 121U/L, *P* = 0.160) and bilirubin (mean 0.83 mg/dL vs. 0.58 mg/dL, *P* = 0.344) as compared to patients with normal VNCa-AV (group 2) (Table 4). In comparison to group 1, mean values for ISM patients (group 3) were: BM MC infiltration 16% vs. 45% (*P* = 0.004), serum tryptase 87 µg/L vs. 437 µg/L (*P* = 0.003) and alkaline phosphatase 88 U/L vs. 339 U/L (*P* = 0.109).

**Table 4:** Disease characteristics of 18 patients with systemic mastocytosis stratified according the virtual-non-calcium attenuation values.

Characteristics	(#1) Increased VNCa values in SSM/AdvSM (n = 8)	(#2) Normal VNCa values in SSM/AdvSM (n = 4)	(#3) Normal VNCa values in ISM/SSM/AdvSM (n = 10)	*P* (#1) vs. (#2)	*P* (#1) vs. (#3)
Hemoglobin, g/dL (mean ± SD)	11.6 ± 1.5	9.5 ± 2.7	11.7 ± 3.0	n.s.	n.s.
Platelets, × 10^9^/L (mean ± SD)	158 ± 72	203 ± 150	254 ± 105	n.s.	0.036
Serum tryptase, µg/L (mean ± SD)	437 ± 200	143 ± 40	87 ± 68	0.008	0.003
Albumin, g/dL (mean ± SD)	38 ± 3.8	37.4 ± . 2.6	40.0 ± 4.5	n.s.	n.s.
Alkaline phosphatase, U/L (mean ± SD)	339 ± 370	121 ± 65	88 ± 48	n.s.	n.s.
Bilirubin, mg/dL (mean ± SD)	0.83 ± 0.5	0.58 ± 0.25	0.56 ± 0.31	n.s.	n.s.
Mast cell infiltration in BM, % (mean ± SD)	45 ± 20	25 ± 15	16 ± 12	n.s.	0.004
*KIT* D816V EAB in PB, % (mean ± SD)	25 ± 18	12 ± 20	10 ± 14	n.s.	n.s.
*KIT* D816V EAB in BM, % (mean ± SD)	26 ± 23	19 ± 16	13 ± 12	n.s.	n.s.

All patients with increased VNCa values were diagnosed as SSM/AdvSM.

*AdvSM* advanced systemic mastocytosis, *BM* bone marrow, *ISM* indolent systemic mastocytosis, *n.s.* not. Significant, *PB* peripheral blood, *SD* standard deviation, *SSM* smoldering systemic mastocytosis, *VNCa* virtual-non-calcium attenuation.

## Discussion

Non-invasive imaging techniques such as CT and MRI are important supplementary tools in the diagnostic work-up of SM. Beside the visualization of potential involvement of visceral organs through organomegaly, this particularly includes qualitative and quantitative assessment of bone lesions such as osteopenia, osteoporosis, osteosclerosis and osteoporotic/osteolytic lesions with pathologic fractures^19^. The correlation between HU values and BM MC infiltration has been established in non-DECT examinations^18^. While osteopenia and osteoporosis are typically present in ISM, osteosclerosis is more frequently identified in AdvSM^26,27^. We recently showed that an increased BMD is strongly associated with advanced disease and inferior outcome^19^. However, conventional CT technology does not allow visualization and quantification of BM edema, which is frequently identified in patients with SSM/AdvSM by MRI^20^.

A recent study successfully applied VNCa from DECT in patients with multiple myeloma. Kosmala et al. showed, that the VNCa-AV significantly differed according to the BM infiltration pattern on MRI and that a diffuse infiltration pattern could be confidently determined using DECT^23^.

In patients with SSM/AdvSM diffuse BM infiltration patterns have also been described using MRI technique^20^, however no data is available about the applicability of DECT in these patients. We therefore evaluated DECT-generated VNCa-AV in clinically and morphologically well characterized patients with ISM and SSM/AdvSM. VNCa-VA were strongly correlated with quantitative bone marrow MC infiltration and serum tryptase levels but also other characteristics, e.g. levels of alkaline phosphatase, albumin or *KIT* D816V variant allele frequency, indicating a strong correlation with an advanced phenotype. Mean VNCa-AV were significantly higher in patients with SSM/AdvSM with values >  − 30HU almost exclusively and values >  − 10HU exlusively found in SSM/AdvSM. Within the SSM/AdvSM patient group, patients with an elevated VNCa-AV revealed a more aggressive phenotype. Of note, two AdvSM patients with regular bone structure by conventional CT showed markedly elevated VNCa-AV. Both patients were associated with a poor clinico-genetic risk profile, e.g. *KIT* D816V negative MCL and multimutated SM-MDS/MPNu progressing to secondary acute myeloid leukemia even more indicating DECT as a supplementary tool for identification of high-risk disease.

The increased VNCa-AV may be best explained by variable displacement of healthy adipose marrow by tumor cells or rather MCs. Because an AHN is present in 70–80% of AdvSM patients, it could be argued that the AHN also contributes to the pathologically elevated VNCa-AV, e.g. through hypercellularity. However, the strong association between VNCa-AV and several SM-specific factors outside of the bone marrow such as serum tryptase levels and to a lesser extent alkaline phosphatase, albumin and *KIT* D816V are clearly in favor of SM.

Several limitations of this study have to be addressed. First, the control group consisted of patients with non-metastatic malignant melanoma and not healthy probands. This patient group was chosen, because (i) the retrospective design of this study did not allow to enroll healthy probands, (ii) scans for metastatic melanomatous deposits in patients with malignant melanoma were mandatory and (iii) the absence of metastases in patients with malignant melanoma and otherwiese “healthy” BM comprised a control group similar to what we expect in a general sex- and age-matched population. Further, the small sample size of 18 patients derived from a single center limits statistical power, especially for subgroup analysis. However, SM is a very rare disease and the application of DECT is not yet part of the routine clinical work-up. Larger patient populations and a prospective study design would be desirable.

Last, to underline our results, the correlation of VNCa-AV with the BM morphology on MRI might be of interest. Unfortunately, due to the retrospective study design timely spine MRI was not available for the patients included in this study. This aspect should also be highlighted in future studies.

Imaging techniques such as DECT are appreciated for its remarkable value in biological characterization of tissue involvement through generating functional information upon the tissue microstructure. Radiological biomarkers, which allow a quantitative and objective analysis of these images, are of increasing importance in this context. We conclude, that DECT represents an excellent supplementary tool for one-stop-shop imaging of organomegaly, osteopenia, osteoporosis, osteosclerosis and bone marrow edema in patients with SM. The technique allows non-invasive assessment of the mast cell burden and may therefore serve as supplementary tool for diagnosis, subclassification and monitoring of the SM disease course.

## Data Availability

The datasets used and/or analysed during the current study are available from the corresponding author on reasonable request.

## Abbreviations

AdvSM: Advanced systemic mastocytosis
AHN: Associated hematologic neoplasm
ASM: Aggressive systemic mastocytosis
BM: Bone marrow
BMD: Bone mineral density
CT: Computed tomography
DECT: Dual-energy computed tomography
ISM: Indolent systemic mastocytosis
MC: Mast cell
MCL: Mast cell leukemia
MDS/MPNu: Myelodysplastic/myeloproliferative neoplasm unclassified
MRI: Magnetic resonance imaging
ROI: Region of interest
SM: Systemic mastocytosis
SM-AHN: Systemic mastocytosis with an associated hematologic neoplasm
SSM: Smoldering systemic mastocytosis
VNCa-AV: Virtual-non-calcium attenuation values
